# Investigation of a UPR-Related Gene Signature Identifies the Pro-Fibrotic Effects of Thrombospondin-1 by Activating CD47/ROS/Endoplasmic Reticulum Stress Pathway in Lung Fibroblasts

**DOI:** 10.3390/antiox12122024

**Published:** 2023-11-21

**Authors:** Jun-Hui Zhan, Juan Wei, Lin Liu, Yi-Tong Xu, Hui Ji, Chang-Nan Wang, Yu-Jian Liu, Xiao-Yan Zhu

**Affiliations:** 1 School of Kinesiology, The Key Laboratory of Exercise and Health Sciences of Ministry of Education, Shanghai University of Sport, Shanghai 200438, China; 2121517019@sus.edu.cn (J.-H.Z.); 2011516009@sus.edu.cn (J.W.); xuyitong@sus.edu.cn (Y.-T.X.); 2021518012@sus.edu.cn (H.J.); 2 School of Sports and Health, Nanjing Sport Institute, Nanjing 210014, China; 9120180024@nsi.edu.cn; 3 Department of Physiology, Navy Medical University, Shanghai 200433, China; wangchangnan@shu.edu.can

**Keywords:** TSP-1, ER stress, ROS, fibroblast, pulmonary fibrosis

## Abstract

Unfolded protein response (UPR) signaling and endoplasmic reticulum (ER) stress have been linked to pulmonary fibrosis. However, the relationship between UPR status and pulmonary function and prognosis in idiopathic pulmonary fibrosis (IPF) patients remains largely unknown. Through a series of bioinformatics analyses, we established a correlation between UPR status and pulmonary function in IPF patients. Furthermore, thrombospondin-1 (TSP-1) was identified as a potential biomarker for prognostic evaluation in IPF patients. By utilizing both bulk RNA profiling and single-cell RNA sequencing data, we demonstrated the upregulation of TSP-1 in lung fibroblasts during pulmonary fibrosis. Gene set enrichment analysis (GSEA) results indicated a positive association between TSP-1 expression and gene sets related to the reactive oxygen species (ROS) pathway in lung fibroblasts. TSP-1 overexpression alone induced mild ER stress and pulmonary fibrosis, and it even exacerbated bleomycin-induced ER stress and pulmonary fibrosis. Mechanistically, TSP-1 promoted ER stress and fibroblast activation through CD47-dependent ROS production. Treatment with either TSP-1 inhibitor or CD47 inhibitor significantly attenuated BLM-induced ER stress and pulmonary fibrosis. Collectively, these findings suggest that the elevation of TSP-1 during pulmonary fibrosis is not merely a biomarker but likely plays a pathogenic role in the fibrotic changes in the lung.

## 1. Introduction

Idiopathic pulmonary fibrosis (IPF) is an age-related interstitial lung disease. It is characterized by the progressive formation of scar tissue in the lungs, leading to impaired gas exchange and eventual respiratory failure [1]. The development of IPF is attributed to a failure in the regeneration of alveolar epithelial cells and an abnormal wound healing response in the presence of pro-fibrotic factors [2,3]. Despite significant progress in understanding the mechanisms underlying this disease, current treatment modalities still have limitations [4]. Therefore, gaining a better understanding of the pathogenesis of IPF is crucial for the development of more effective and safer treatments.

The endoplasmic reticulum (ER) is a highly versatile protein factory equipped with chaperones and enzymes essential for proper protein folding and quality control in the ER. Any condition that disrupts protein processing can lead to the accumulation of misfolded proteins in the ER, resulting in ER stress and the activation of the unfolded protein response (UPR) [5]. Accumulating basic studies have linked ER stress and UPR signaling to the development of pulmonary fibrosis. Specifically, the reactive oxygen species (ROS)-mediated UPR/ER stress has been shown to promote epithelial–mesenchymal transition and the differentiation of fibroblasts to myofibroblasts, which are key effector cells in fibrotic diseases and produce excessive amounts of collagen and other components of the extracellular matrix (ECM) [6,7,8]. However, the relationship between ER stress/UPR status and pulmonary function and prognosis in patient with IPF remains largely unknown.

Thrombospondin-1 (TSP-1), which is encoded by the THBS1 gene, is a secreted matricellular glycoprotein that plays diverse and significant roles in tissue remodeling [9,10]. Previous studies have provided evidence highlighting the importance of TSP-1 in controlling the activation of latent transforming growth factor (TGF-β) in fibrotic renal disease and diabetic complications [11,12]. Additionally, TSP-1 has been identified as a damage controller in the context of lung inflammation and repair, both in noninfectious [13,14] and infectious [15,16,17] lung injury models. However, little is known about the role of TSP-1 in the pathogenesis of pulmonary fibrosis.

Through a series of bioinformatics analyses, we established a correlation between UPR status and pulmonary function in IPF. Furthermore, our investigation into UPR-related gene signatures identified TSP-1 as a potential biomarker for prognostic evaluation in IPF patients. Through a series of bioinformatics analyses, we demonstrated, for the first time, the upregulation of TSP-1 in lung fibroblasts during pulmonary fibrosis. Gene set enrichment analysis (GSEA) results indicated a positive association between TSP-1 expression and gene sets related to ROS and UPR signaling pathways. Consequently, this study shed light on the impact of TSP-1 overexpression and inhibition on the development of pulmonary fibrosis, and further investigated the underlying mechanisms.

## 2. Materials and Methods

### 2.1. The Administration of Bleomycin and Drug Treatment

After anesthesia, the mice were intratracheally instilled with bleomycin (Selleck Chemicals, Houston, TX, USA) at a dose of 1.5 mg/kg in a total volume of 50 µL [18]. Control mice received only 50 µL of sterile PBS via endotracheal instillation. The TSP-1 inhibitor leucine-serine-lysine-leucine (LSKL. MCE, Monmouth Junction, NJ, USA) and CD47 inhibitor RRx-001 (MCE, NJ, USA) were prepared to the appropriate concentrations and stored at −80 °C for subsequent experiments after sequential addition of 10% DMSO and 90% corn oil according to the reagent vendor’s requirements.

To investigate the role of LSKL, RRx-001, and intrapulmonary TSP-1 overexpression on bleomycin-induced lung fibrosis, mice were randomly divided into the following groups [19,20,21,22,23]: (1) Control group; (2) LSKL group; (3) RRx-001 group; (4) Bleomycin Group; (5) Bleomycin and LSKL group; (6) Bleomycin and RRx-001 group; (7) Lv-Control group; (8) Lv-TSP-1 group; (9) Lv-Control + bleomycin group; (10) Lv-TSP-1 + bleomycin group. Lung samples were collected 14 days after bleomycin or PBS instillation. Details are provided in the Supplementary Materials.

### 2.2. Masson’s Trichrome Staining

Collagen fiber formation was detected using Masson trichrome staining as previously described [24,25].

### 2.3. The Determination of Hydroxyproline Content

Fresh lung tissues were extracted and stored at −80 °C. Hydroxyproline (HYP) Assay Kit (Jiancheng, Nanjing, China) was used for the determination of hydroxyproline content in lung tissues according to the manufacturer’s instruction [26].

### 2.4. The Analysis of Publicly Available Gene Expression Data and High Throughput Sequencing Data

The GSE32537 dataset [27] was obtained and downloaded from the Gene Expression Omnibus (GEO, http://www.ncbi.nlm.nih.gov/geo/, accessed on 26 October 2022), containing 119 IPF samples. The sequencing samples were obtained from the patients’ lung tissue, and the clinical features of GSE32537 include percent predicted forced vital capacity (FVC% predicted), percent predicted diffusion capacity of the lung for carbon monoxide (DLCO% predicted), and the St. George’s Respiratory Questionnaire (SGRQ) scores. Additionally, 19 additional datasets (GSE27957 [28], GSE93606 [29], GSE38958 [30], GSE47460 [31], GSE135893 [32], GSE132771 [33], GSE201698, GSE129605 [34], GSE119007 [35], GSE103488 [36], GSE135097 [37], GSE226249 [38], GSE44723 [39], GSE172121 [40], GSE161322 [41], GSE97829 [42], GSE102674 [43], GSE130983 [44], GSE97826 [42]) were used for external validation of the analysis results.

The discovery dataset GSE32537 was first normalized using the limma (v.3.52.4) package. The correspondence between probes and gene symbols was extracted from the GPL6244 Platforms. Multiple probes corresponding to the same gene symbol were removed by averaging the highest expression levels.

Four single-cell RNA sequencing datasets were obtained from GEO datasets and analyzed using the R package “Seurat”. After reading the data and performing quality control, the SCTransform function was used to standardize and scale the data. If there were multiple datasets, the harmony package was used to integrate the data. Dimension reduction was performed using the RunUMAP() function. Seurat’s FindMarkers() and FindClusters() functions were used to identify markers and DEGs. Cell types were labeled based on CellMarker (http://xteam.xbio.top/CellMarker/search.jsp, accessed on 14 November 2022) and the “SingleR” package.

This study utilized twenty publicly available GEO datasets with preexisting ethics approval from original studies. Patients’ informed consents were available in these twenty public databases.

### 2.5. The Identification of UPR Status and DEGs

The reference gene sets for the unfolded protein response (UPR) were downloaded from the Molecular Signatures Database (MSigDB, HALLMARK_UNFOLDED_PROTEIN_RESPONSE.v2022.1.Hs.json) which includes 113 genes. The Uniform Manifold Approximation and Projection (UMAP) algorithm was applied to divide the samples into two clusters. Based on these clusters, two groups, “UPR^high^” and “UPR^low^”, were identified. Details are provided in the Supplementary Materials.

### 2.6. Correlation Analysis and LASSO Regression

The “Hmisc” and “glmnet” packages were used for correlation analysis and the Least Absolute Shrinkage and Selection Operator (LASSO) regression, respectively. Based on the expression values of the UPR-related DEGs, correlation analysis and LASSO regression were performed with clinical data to further screen UPR-related DEGs. The multi-response Gaussian family was used to handle a number of (correlated) responses, where a variable is either included in the model for all the responses or excluded for all the responses. Ten-fold cross-validation and 1000 iterations were conducted to reduce potential instability in the results. The value of λ that minimizes the standard error during the construction of the regression equation was chosen [45].

### 2.7. Functional Analysis and Survival Analysis

Gene set enrichment analysis (GSEA) was performed using the GSEA software v4.2.1 (https://www.gsea-msigdb.org/gsea/index.jsp, accessed on 4 January 2022) [46] with all expressed genes. The Gene Set Variation Analysis (GSVA), a nonparametric unsupervised analysis method, was performed using the GSVA package [47]. The gene sets HALLMARK, GO, and REACTOME were selected as the reference gene set and downloaded from MSigDB. Gene sets with a normal *p*-value < 0.05 were considered significantly enriched.

Survival data for each cohort was obtained using the “GEOquery” package. Kaplan–Meier analysis with the log-rank test was performed between groups using the R package “survival”. The “survminer” package was used to identify the optimal cutoff point for dividing samples based on the corresponding survival data.

### 2.8. Quantitative Polymerase Chain Reaction (qPCR)

Total RNA was extracted from tissues and cells using TRIzol reagent (Invitrogen, Carlsbad, CA, USA). The primer sequences are provided in Supplementary Table S1. Details are provided in the Supplementary Materials.

### 2.9. Cell Culture and Stable Transfection

Mouse lung fibroblast Mlg cells were transfected with Lv-TSP-1 to generate a stable TSP-1-overexpressing cell line [48]. Details are provided in the Supplementary Materials.

### 2.10. Immunofluorescence and Immunohistochemistry Staining

To assess ROS production in Mlg and lung tissue, a dihydroethidium (DHE) fluorescent dye probe was used. For the assessment of ROS production in Mlg, cells were seeded on coverslips and incubated with freshly prepared DHE at a dose of 10 µM for 30 min in the dark. Nuclei were stained with blue using 4′,6-diamidino-2-phenylindole (DAPI) [49].

To assess ROS production in lung tissue, fresh lung tissue was fixed in a 4% paraformaldehyde solution for 24 h. Dehydration was performed using 15% and 30% sucrose solutions for 15 min each. The tissue was then embedded in Tissue-Tek OCT compound, snap frozen, cryosected at a thickness of 5 μm, and collected on Superfrost plus slides. Cryosections of lung tissues were then stained with 10 µM DHE in a dark humid environment for 30 min, and the nuclei were stained with DAPI [50].

For immunofluorescence staining of macrophage marker F4/80, sections of mouse lung tissue were taken from paraffin-embedded tissue. The samples were first subjected to standard deparaffinization techniques and antigenic retrieval by microwave. After blocking with 3% bovine serum albumin for 30 min, sections were incubated overnight at 4 °C with primary antibodies against F4/80 (Servicebio, Wuhan, China). Next, lung sections were incubated with secondary antibodies conjugated with Alexa Fluor^®^ 488 for 1 h in the dark. Nuclei were counterstained with DAPI. Fluorescence image acquisition was performed using a Nikon fluorescent microscope after slide mounting.

For immunohistochemistry (IHC) staining of TSP-1, 5 µm sections were deparaffinized and rehydrated. After the antigen was retrieved by microwave heating of histologic sections, lung sections were incubated with the primary antibodies against TSP-1 at 4 °C overnight and with horseradish peroxidase-labeled secondary antibodies (Proteintech, Wuhan, China) at room temperature for 50 min, followed by 3,3′-diaminobenzidine (DAB) color development and hematoxylin counterstain (Servicebio, Wuhan, China). For negative controls, the primary antibody was substituted with a mouse IgG in the same dilution.

### 2.11. Western Blot

Details are provided in the Supplementary Materials.

### 2.12. Statistical Analysis

**Figure 1:**
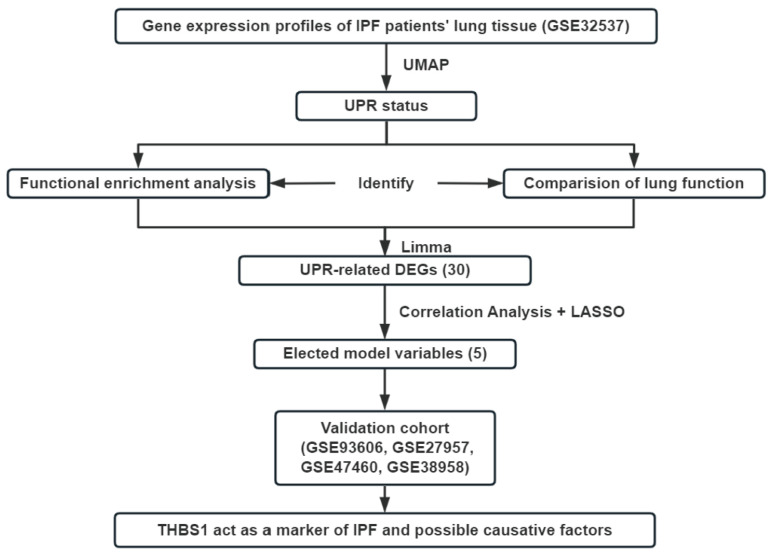
The general idea and methodologies used in this study.

The R software (version 4.2.0) and corresponding packages were used for the bioinformatic analysis. The general idea and methodologies used in the bioinformatic analysis are shown in a flow chart (Figure 1). The packages “ggplot2” and “pheatmap” were utilized to optimize data presentation. The data were presented as means ± SEM. A *p* value of <0.05 was considered significant. Details are provided in the Supplementary Materials.

## 3. Results

### 3.1. A High UPR Status Is Associated with Poorer Pulmonary Function in Patients with IPF

**Figure 2:**
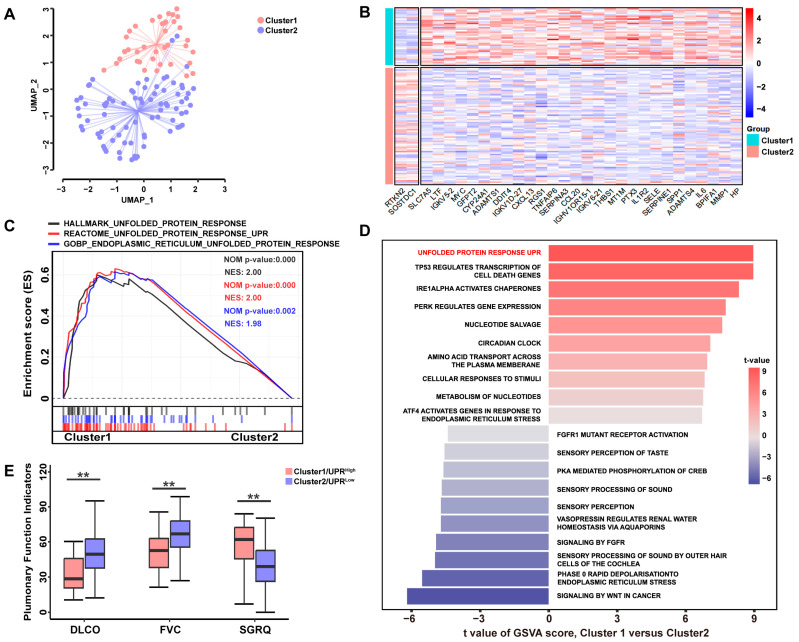
A high UPR status is associated with poorer pulmonary function in patients with IPF. (**A**) UMAP clustering plot based on a marker gene set of the unfolded protein response (UPR). (**B**) Heatmap showing the differential gene expression profiles between Cluster 1 and Cluster 2. (**C**) Following GSEA analysis, the pathway of the UPR, based on Hallmark, Reactome, and GO biological process, was found to be significantly enriched in Cluster 1 patients. (**D**) GSVA-derived histogram of pathways of two clusters, the UPR pathway was most enriched in Cluster 1. Intercept is the top ten most significant pathways in the upward and downward regions, respectively. (**E**) The box plot showed a significant difference in pulmonary lung indices between UPR^high^ and UPR^low^ groups. ** *p* < 0.01.

The discovery cohort consisted of 119 IPF patients obtained from the GEO database GSE32537, and their clinical information is presented in Supplementary Table S2. Using an expression matrix constructed by a set of 113 UPR marker genes from MSigDB, the non-linear dimensionality reduction algorithm UMAP was used to determine two clusters. Each patient was assigned to the nearest cluster (Figure 2A). Cluster 1 comprised 39 patients, while Cluster 2 contained 80 patients. By comparing the expression profiles between the two clusters, we identified 30 DEGs associated with UPR (Figure 2B). GSEA revealed that genes overexpressed in Cluster 1 were enriched in “Hallmark unfolded protein response (Systematic name:M5922)”, “Reactome unfolded protein response UPR (Systematic name:M10294)”, and “GOBP endoplasmic reticulum unfolded protein response (Systematic name:M22993)” (Figure 2C, Supplementary Figure S1A). Similar conclusions were obtained from GSVA (Figure 2D), and UPR signaling was found to be common among the top three gene sets identified using both GSEA and GSVA. These findings indicated that Cluster 1 exhibited a high level of UPR. Consequently, patients in Cluster 1 and Cluster 2 were classified as the UPR^high^ and UPR^low^ groups, respectively. Among the 30 DEGs, 28 were found to be overexpressed in the UPR^high^ cluster. The clinical information for each cluster is presented in Supplementary Table S3. Furthermore, the clinicopathologic features of the UPR^high^ and UPR^low^ groups were further analyzed. As shown in Figure 2E, the patients in the UPR^low^ group generally exhibited higher carbon monoxide diffusing capacity (DLCO) (*p* < 0.001) and forced vital capacity (FVC) (*p* < 0.0001), indicating better lung function. Conversely, patients in the UPR^high^ group had higher scores on the St George’s Respiratory Questionnaire (SGRQ) (*p* < 0.01), suggesting worse lung function and quality of life. These findings suggest a potential correlation between a high UPR status and poorer pulmonary function in patients with IPF.

### 3.2. UPR-Related Gene Signature Predicts the Pro-Fibrotic Effects of TSP-1

**Figure 3:**
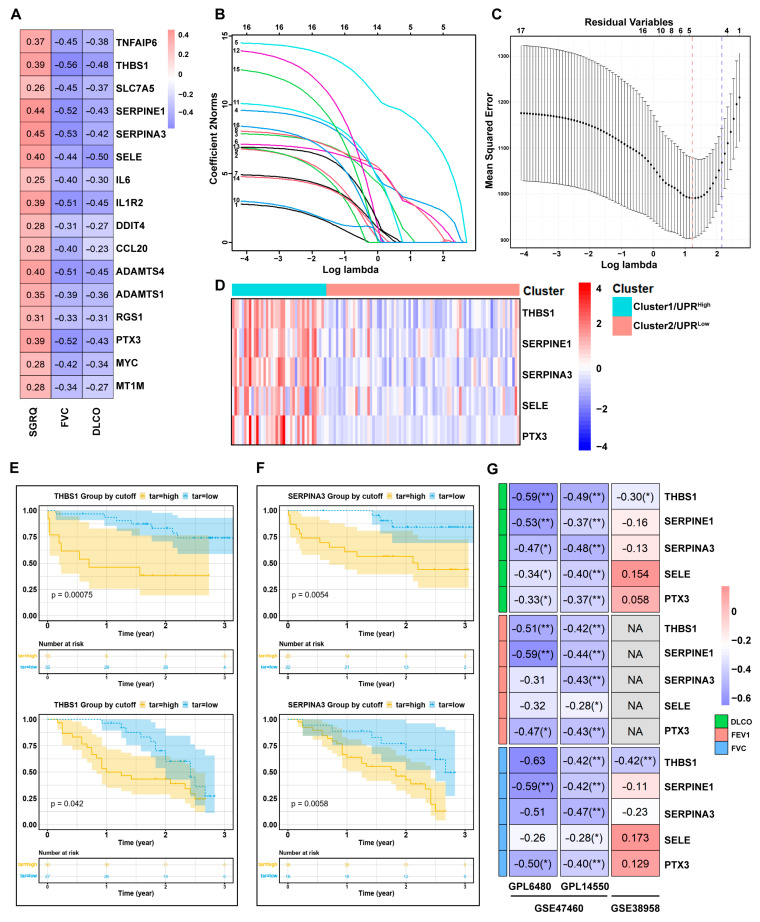
UPR-related gene signature predicting the pro-fibrotic effects of TSP-1. (**A**) Heatmap showing the significant correlation between three clinicopathologic features and 16 UPR-related DEGs. (**B**) LASSO coefficient profiles of 16 screened UPR-related DEGs. (**C**) Ten-fold cross-validation of LASSO analysis. Error bars represent the SE. The dotted vertical lines show the optimal values. Red dotted line represents lambda.min which gives minimum mean cross-validated error, while blue dotted line represents lambda.1se that gives the most regularized model. (**D**) Heatmap showing the expression profiles of five optimal UPR-related DEGs between UPR^high^ and UPR^low^ groups. (**E**) Kaplan–Meier plot of overall survival between high level THBS1 and low level THBS1 expression in IPF cohorts of GSE27957 (top) and GSE93606 (bottom). The cutoff value is selected using the survminer package. (**F**) Kaplan–Meier plot of overall survival between high level SERPINA3 and low level SERPINA3 expression in IPF cohorts of GSE27957 (top) and GSE93606 (bottom). The cutoff value is selected using the survminer package. (**G**) Heatmap showing the correlations between five optimal UPR-related DEGs and three clinicopathologic features in IPF cohorts of GSE47460 (platforms GPL6480 and GPL14550) and GSE38958. NA, data not available. * *p* < 0.05; ** *p* < 0.01.

To assess the relationship between 30 UPR-related DEGs and clinicopathologic features, Pearson correlation analysis was conducted for each DEG. As shown in Figure 3A and Supplemental Table S4, a total of 16 UPR-related DEGs (TNFAIP6, THBS1, SLC7A5, SERPINE1, SERPINA3, SELE, RGS1, PTX3, MYC, MT1M, IL6, IL1R2, DDIT4, CCL20, ADAMTS4, ADAMTS1) exhibited significant correlations with all three clinicopathologic features (*p* < 0.05). These 16 UPR-related DEGs displayed negative correlations with FVC and DLCO, while positively correlating with SGRQ scores.

To identify the UPR-related DEGs that best accounted for the clinicopathologic phenotype in IPF patients, LASSO regression with 10-fold cross-validation was employed. Figure 3B,C illustrated that five optimal variables (THBS1, SERPINE1, SERPINA3, SELE, PTX3) were selected from the aforementioned 16 UPR-related DEGs (with an optimal sparseness parameter λ of 9.51). The expression profiles of these five optimal DEGs between UPR^high^ and UPR^low^ groups were visualized in a heatmap (Figure 3D).

Next, the association of the five optimal UPR-related DEGs with the survival status of IPF patients was verified using external datasets “GSE93606” and “GSE27957”. As shown in Figure 3E and Supplemental Figure S2A,B, higher expression of THBS1 or SERPINA3 was associated with a significantly poorer prognosis in both the GSE27957 and GSE93606 IPF cohorts.

Furthermore, the correlation between the five optimal UPR-related DEGs and clinicopathologic features of IPF patients was further analyzed in external datasets “GSE38958” and “GSE47460”. Supplementary Table S5 showed the demographics of human patients in the validation cohort. It was observed that only THBS1 exhibited a significant correlation with lung function indices DLCO, FVC, and FEV1 (forced expiratory volume in one second) in IPF cohorts of both GSE38958 and GSE47460. SERPINE1 and PTX3 showed significant correlations with DLCO, FVC, and FEV1 in IPF cohorts of GSE47460, but did not exhibit significant correlations with DLCO and FVC in IPF cohorts of GSE38958 (Figure 3F).

Taken together, these findings suggest that TSP-1 may have a pro-fibrotic effect in the lungs, and higher TSP-1 expression may serve as a predictor of worse lung function and prognosis in IPF patients.

### 3.3. The Effect of Intrapulmonary TSP1 Overexpression on the Development of ER Stress and Pulmonary Fibrosis in Mice

**Figure 4:**
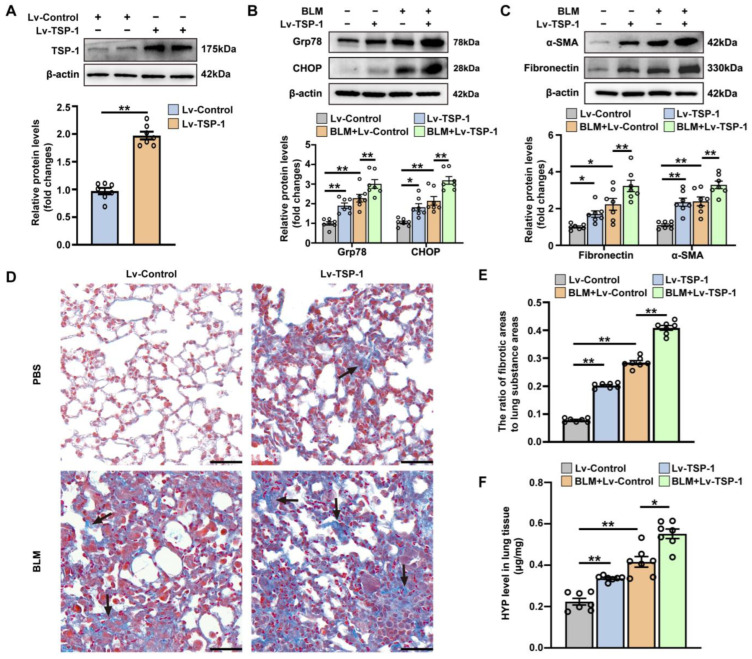
The effect of intrapulmonary TSP1 overexpression on the development of ER stress and pulmonary fibrosis in mice. (**A**) Mice were intratracheally instilled with Lv-TSP-1 or Lv-Control. Two weeks later, lung tissues were harvested to determine TSP-1 expression by Western blot analysis. Representative protein bands are presented at the top of the histograms. (**B**–**F**) ICR mice were randomized into four groups: Lv-Control, Lv-TSP-1, Lv-Control + BLM, and Lv-TSP-1 + BLM. (**B**) Protein levels of Grp78 and CHOP in lung. Representative protein bands are presented at the top of the histograms. (**C**) Protein levels of fibronectin and α-SMA in lung tissues. Representative protein bands are presented at the top of the histograms. (**D**) Masson’s trichrome staining. Black arrows point to areas of collagen fiber deposition. Scale bars correspond to 50 μm. (**E**) Quantification of the ratio of collagen-deposited areas to lung substance areas (a morphometric measure of pulmonary fibrosis). (**F**) Pulmonary hydroxyproline (HYP) levels. Data are expressed as mean ± SEM (n = 7). * *p* < 0.05; ** *p* < 0.01. BLM represents bleomycin.

The THBS1 gene encodes the glycoprotein TSP-1. To assess the effects of ectopic TSP-1 overexpression, we constructed a lentiviral vector (Lv-TSP-1) that expressed TSP-1 and administered it to mice treated with either PBS or bleomycin. As shown in Figure 4A, intratracheal instillation of Lv-TSP-1 resulted in an approximately twofold increase in pulmonary TSP-1 expression. Notably, intrapulmonary TSP-1 overexpression alone significantly elevated the protein levels of ER stress-related proteins Grp78 and the downstream transcription factor CHOP in lung tissues (Figure 4B). Moreover, Lv-TSP-1-injected mice exhibited increased pulmonary expression of fibrosis markers α-SMA and fibronectin compared to Lv-Control-injected mice (Figure 4C). Masson’s trichrome staining revealed a mild but significant collagen deposition in lung sections of mice with intrapulmonary TSP-1 overexpression (Figure 4D,E). Additionally, Lv-TSP-1-injected mice displayed potentiated bleomycin-induced ER stress and lung fibrosis, characterized by heightened expression of ER stress and fibrosis markers, increased collagen deposition, and more severe disruption of normal pulmonary architecture. To quantify the collagen deposition in the lung, we analyzed changes in hydroxyproline levels. Figure 4F demonstrated that both Lv-TSP-1 overexpression and bleomycin treatment alone led to increased pulmonary hydroxyproline content. In bleomycin-treated mice, the Lv-TSP-1-injected group exhibited significantly higher pulmonary hydroxyproline levels compared to the Lv-Control-injected group. Therefore, TSP-1 overexpression alone is sufficient to induce mild ER stress and pulmonary fibrosis, and it can even exacerbate bleomycin-induced ER stress and pulmonary fibrosis. These findings suggest that the elevation of TSP-1 during pulmonary fibrosis is not merely a biomarker but likely pathogenic for the fibrotic changes in the lung.

Interstitial macrophage accumulation has been recognized as an important contributor to the pathogenesis of pulmonary fibrosis [1,5]. We then investigated the impact of intrapulmonary TSP-1 overexpression on macrophage infiltration in bleomycin-treated mice. As shown in Supplementary Figure S3, lung tissues obtained from both Lv-Control-injected mice and Lv-TSP-1-injected mice exhibited very few F4/80 positive staining macrophages. However, in the case of bleomycin-treated mice, there was a severe infiltration of F4/80 positive staining macrophages in the lung tissues, and this infiltration was not significantly affected by intrapulmonary TSP-1 overexpression. These findings suggest that the pro-fibrotic effects of TSP-1 may be independent of its effects on macrophage infiltration.

### 3.4. TSP-1 Inhibitor Attenuates Bleomycin-Induced ER Stress and Pulmonary Fibrosis

**Figure 5:**
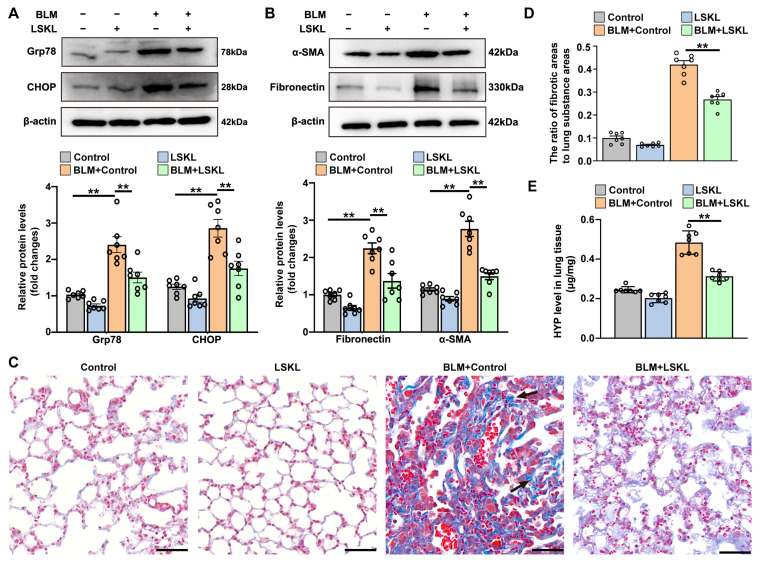
TSP-1 inhibitor attenuates bleomycin-induced ER stress and pulmonary fibrosis. ICR mice were randomized into four groups: Control, LSKL, BLM + Control and BLM + LSKL. (**A**) Protein levels of Grp78 and CHOP in lung tissues. (**B**) Protein levels of α-SMA and fibronectin in lung tissues. (**C**) Representative images of Masson’s trichrome staining of lung fibrosis. Black arrows point to areas of collagen fiber deposition. Scale bars correspond to 50 μm. (**D**) Quantification of the ratio of collagen-deposited areas to lung substance areas (a morphometric measure of pulmonary fibrosis). (**E**) Pulmonary hydroxyproline (HYP) levels. Data are expressed as mean ± SEM (n = 7). ** *p* < 0.01. BLM represents bleomycin.

We then investigated the impact of the TSP-1 inhibitor leucine-serine-lysine-leucine (LSKL) on bleomycin-induced ER stress and pulmonary fibrosis. Figure 5A,B demonstrated that treatment with LSKL mitigated the bleomycin-induced upregulation of Grp78, CHOP, α-SMA, and fibronectin in lung tissues. Masson’s trichrome staining revealed that LSKL treatment markedly reduced collagen deposition and preserved the normal pulmonary architecture in bleomycin-treated mice (Figure 5C,D). LSKL also significantly decreased the bleomycin-induced elevation of pulmonary hydroxyproline levels (Figure 5E). These findings collectively indicate that administration of the TSP-1 inhibitor can effectively attenuate ER stress and pulmonary fibrosis in mice exposed to bleomycin.

### 3.5. TSP-1 Is Upregulated in Lung Fibroblasts during Pulmonary Fibrosis

**Figure 6:**
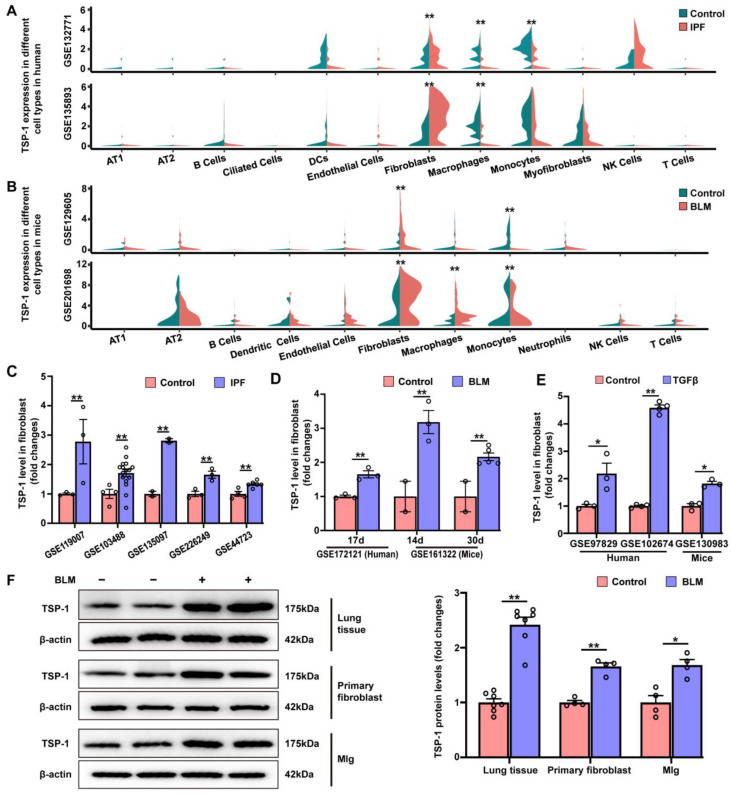
TSP-1 is upregulated in lung fibroblasts during pulmonary fibrosis. (**A**,**B**) Single-cell RNA sequencing demonstrated the expression profile of TSP-1 in major cell types detected in humans (**A**) and mice (**B**). (**C**) Published GEO datasets were analyzed for TSP-1 expression in lung fibroblasts isolated from IPF patients. (**D**) Published GEO datasets were analyzed for TSP-1 expression in lung fibroblasts treated with bleomycin in vitro for the indicated time periods. (**E**) Published GEO datasets were analyzed for TSP-1 expression in primary cultured lung fibroblasts treated with TGF-β in vitro. (**F**) Mice were intratracheally instilled with bleomycin (1.5 mg/kg) or PBS. Lung tissues or fibroblasts were obtained at day 14 after bleomycin instillation. The mouse lung fibroblast cell line Mlg was treated with bleomycin (5 μg/mL) for 48 h. TSP-1 protein expression in lung tissues, lung fibroblasts, and Mlg cells was assessed by Western blot. Representative protein bands are presented on the left side of corresponding histograms. Lung tissue samples were collected from seven control mice and seven bleomycin-treated mice. Data are expressed as mean ± SEM. * *p* < 0.05; ** *p* < 0.01. BLM represents bleomycin.

To investigate the distribution and expression pattern of TSP-1 in healthy and fibrotic lung tissues, we analyzed transcriptomes of single cells from control individuals and IPF patients using publicly available datasets described by Tsukui et al. (GSE132771) [33] and Habermann et al. (GSE135893) [32]. Our analysis revealed that TSP-1 was predominantly expressed in fibroblasts and immune cells, including macrophages, monocytes, and dendritic cells, in both healthy and fibrotic lung tissues (Figure 6A). Importantly, IPF patients exhibited a significant upregulation of TSP-1 in fibroblasts compared to healthy controls. These findings were further supported by bioinformatics analysis of single-cell RNA sequencing data from murine models of bleomycin-induced pulmonary fibrosis (Figure 6B), which showed similar results to those observed in IPF patients. Notably, TSP-1 expression in macrophages and monocytes exhibited a significant reduction in IPF patients compared to healthy controls (Figure 6A and Supplementary Figure S4A). In murine models of bleomycin-induced pulmonary fibrosis, we observed a profound increase in TSP-1 expression in macrophages following bleomycin treatment, while a decrease in TSP-1 expression was observed in monocytes (Figure 6B and Supplementary Figure S4B).

As depicted in Figure 6C,D, TSP-1 expression was significantly higher in lung fibroblasts from IPF patients or bleomycin-treated mice compared to control individuals or mice, respectively. Additionally, in primary cultured lung fibroblasts treated with TGF-β in vitro, the analysis revealed that TGF-β treatment for 24 h significantly increased TSP-1 expression in both human and murine lung fibroblasts (Figure 6E).

Western blot showed that TSP-1 expression was significantly elevated in both lung tissues and lung fibroblasts of bleomycin-exposed mice compared to control mice (Figure 6F). Furthermore, we observed a significant increase in TSP-1 protein expression in the mouse lung fibroblast cell line Mlg cells following bleomycin treatment. We then conducted IHC staining on lung sections and confirmed that pulmonary TSP-1 expression was significantly elevated in bleomycin-exposed mice compared to control mice (Supplementary Figure S5). Notably, in lung sections of bleomycin-treated mice, TSP-1 positive staining was identified in fibroblasts within fibroblastic foci.

Collectively, the results from the bioinformatic analysis and validation experiments indicate that TSP-1 is upregulated in lung fibroblasts during pulmonary fibrosis.

### 3.6. Stable Overexpression of TSP-1 Promotes Fibroblast Activation by CD47/ROS/ER Stress Signaling Pathway

**Figure 7:**
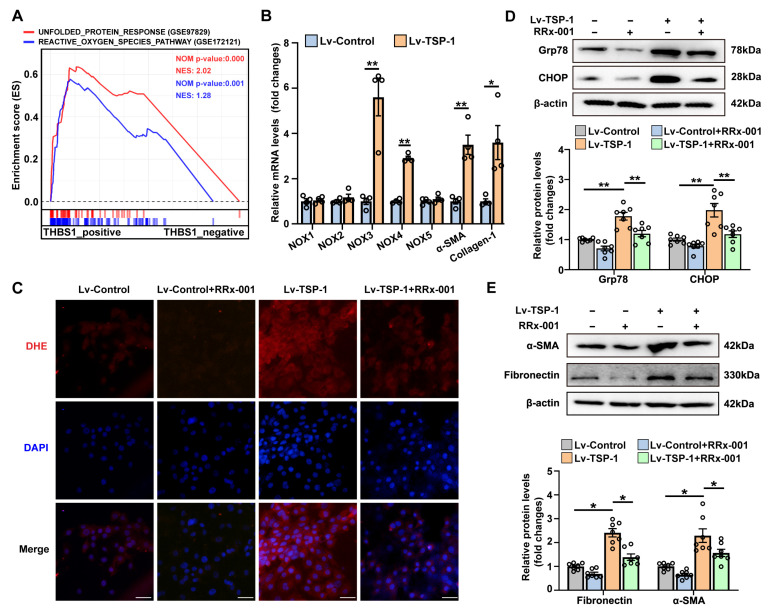
Stable overexpression of TSP-1 promotes fibroblast activation by CD47/ROS/ER stress signaling pathway. (**A**) GSEA analysis showed the positive correlation between THBS1 gene expression and UPR and ROS pathways. (**B**) Mlg cells were transfected with Lv-Control or lentivirus expressing TSP-1 (Lv-TSP-1) to establish a fibroblast cell line with stable TSP-1 overexpression. Expression of NOX family members (NOX1-5) and fibrotic markers (α-SMA and Collagen-1) in TSP-1-overexpressed Mlg cells was assessed by qPCR. (**C**–**E**) Mlg cells stably transfected with Lv-TSP-1 or Lv-Control were treated with vehicle or CD47 inhibitor RRx-001 (5 µM) for 48 h. (**C**) ROS production was examined by dihydroethidium (DHE) staining (red). Nuclei were stained with blue using 4′, 6-diamidino-2-phenylindole (DAPI). Scale bars correspond to 50 μm. (**D**) The protein levels of Grp78 and CHOP were determined by Western blot analysis. Representative protein bands were presented on the top of the histograms. (**E**) The protein levels of α-SMA and fibronectin were determined by Western blot analysis. Representative protein bands are presented at the top of the histograms. Data are expressed as mean ± SEM (n = 7). * *p* < 0.05; ** *p* < 0.01.

To investigate the role of TSP-1 upregulation in fibroblasts, we aimed to identify potential key signaling pathways associated with TSP-1 expression using single-gene GSEA based on RNA profiling data from bleomycin-treated fibroblasts. As anticipated, we observed a positive correlation between TSP-1 expression and gene sets related to the UPR pathway (Figure 7A and Supplementary Figure S6A). Notably, the ROS signaling pathway, known to promote ER stress, also exhibited a significant correlation with TSP-1 expression in fibroblasts (Figure 7A and Supplementary Figure S6A,C). Meijles et al. recently reported that TSP-1 can enhance NADPH oxidase (NOX)-dependent generation of ROS in endothelial cells through its receptor CD47 [51]. Therefore, we proceeded to investigate the impact of stable TSP-1 overexpression on ROS production and ER stress in Mlg cells. Western blot and qPCR confirmed the effectiveness of TSP-1 overexpression in Mlg cells (Supplementary Figure S6D). We observed that stable transfection of Lv-TSP-1 significantly upregulated the mRNA expression of fibrotic markers α-SMA and Collagen-1, as well as NADPH oxidase isoforms NOX3 and NOX4 (Figure 7B). As depicted in Figure 7C and Supplementary Figure S6E, Lv-TSP-1-treated fibroblasts exhibited a significant increase in ROS production, as measured by the fluorescence of DHE. Stable TSP-1 overexpression also markedly increased the protein expression of Grp78 and CHOP in Mlg fibroblasts, indicating activation of ER stress (Figure 7D). Importantly, we found that TSP-1 overexpression-induced ROS production and ER stress were effectively blocked by the CD47 inhibitor RRx-001 (Figure 7C,D and Supplementary Figure S6E). Furthermore, treatment with the CD47 inhibitor significantly attenuated the upregulation of α-SMA and fibronectin protein expression induced by TSP-1 overexpression in Mlg fibroblasts (Figure 7E). These findings suggest that stable overexpression of TSP-1 may promote fibroblast activation through the CD47/ROS/ER stress signaling pathway.

### 3.7. CD47 Inhibitor Attenuates BLM-Induced ROS Production, ER Stress, and Pulmonary Fibrosis

**Figure 8:**
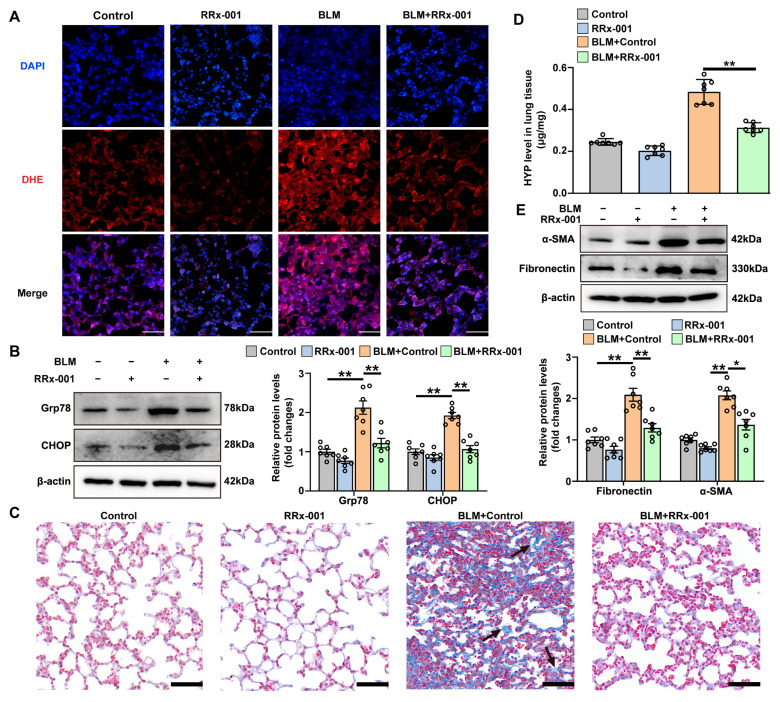
CD47 inhibitor attenuates BLM-induced ROS production, ER stress, and pulmonary fibrosis. ICR mice were randomized into four groups: Control, RRx-001, BLM + Control, and BLM + RRx-001. (**A**) ROS production was examined by DHE staining (red) in lung sections. Nuclei were stained with blue using DAPI (blue). Scale bars correspond to 50 μm. (**B**) The protein levels of Grp78 and CHOP in lung tissues were determined by Western blot analysis. Representative protein bands were presented on the left of the histograms. (**C**) Masson’s trichrome staining. Black arrows point to areas of collagen fiber deposition. Scale bars correspond to 50 μm. (**D**) Pulmonary hydroxyproline (HYP) levels. (**E**) The protein levels of α-SMA and fibronectin in lung tissues were determined by Western blot analysis. Representative protein bands are presented at the top of the histograms. Data are expressed as mean ± SEM (n = 7). * *p* < 0.05; ** *p* < 0.01. BLM represents bleomycin.

In a mouse model of bleomycin-induced pulmonary fibrosis, we observed that treatment with the CD47 inhibitor RRx-001, at a dose of 10 mg/kg for a duration of 2 weeks, significantly reduced ROS production in lung sections of mice exposed to bleomycin, as measured by the fluorescence of DHE (Figure 8A and Supplementary Figure S7A). Additionally, we found that the upregulation of Grp78 and CHOP induced by bleomycin was attenuated by the administration of RRx-001 (Figure 8B). Moreover, RRx-001 treatment demonstrated a significant reduction in collagen deposition and preservation of pulmonary architecture in bleomycin-treated mice (Figure 8C and Supplementary Figure S7B). Consistent with these histological findings, bleomycin-induced increases in pulmonary levels of hydroxyproline, fibronectin, and α-SMA were also significantly attenuated by administration of CD47 inhibitor (Figure 8D,E). These results indicate that the CD47 inhibitor effectively mitigates ROS production, ER stress, and pulmonary fibrosis in mice exposed to bleomycin.

## 4. Discussion

Patients with IPF experience progressive scarring that leads to stiffened alveoli, reduced lung capacity, and impaired gas exchange. This decline in lung function is believed to be associated with various cellular stress events, including ER stress [52]. In this study, we examined the transcriptional profiles of lung tissues from IPF patients and discovered a correlation between higher UPR status and poorer pulmonary function in these individuals. These findings offer new insights into the relationship between pulmonary biomarkers of ER stress and the stratification of IPF patients.

Through multivariable LASSO regression analysis, we identified the five UPR status-related DEGs (THBS1, SERPINE1, SERPINA3, SELE, PTX3) that exhibited the strongest correlation with the clinicopathologic phenotype in IPF patients. Among these five optimal UPR status-related DEGs, TSP-1 demonstrated a significant association with the lung function indices DLCO, FVC, and forced expiratory volume in one second (FEV1) in both PBMCs (GSE38958) and lung tissue (GSE47460) samples. Furthermore, higher expression of TSP-1 was linked to a significantly worse prognosis in two IPF cohorts (GSE27957, PBMCs; GSE93606, whole blood). These bioinformatic findings align with previous studies that have reported elevated circulating levels of TSP-1 in patients with IPF and idiopathic interstitial pneumonia (IIP) compared to healthy controls [53,54,55]. Additionally, serum TSP-1 levels are inversely correlated with a clinical and functional indicator of disease severity, the percentage of predicted vital capacity (%VC) in patients with IIP (specifically, usual interstitial pneumonia plus non-specific interstitial pneumonia) [53]. Therefore, TSP-1 holds promise as a potential biomarker for assessing disease severity and predicting prognosis in individuals with pulmonary fibrosis.

Furthermore, we observed significant correlations between SERPINE1 and PTX3 levels and the lung function indices DLCO, FVC, and FEV1 in lung tissue samples from two IPF cohorts. However, these correlations were not observed in PBMC samples, nor were SERPINE1 and PTX3 levels associated with prognosis in PBMC and whole blood samples of IPF patients. SERPINE1, also known as plasminogen activator inhibitor 1 (PAI-1), is a serine protease inhibitor that promotes the proliferation and transdifferentiation of lung fibroblasts, as well as cell senescence of AT2 cells during lung fibrosis [56,57]. Pentraxin 3 (PTX3), a soluble pattern recognition molecule belonging to the humoral innate immune system, is primarily produced by fibroblasts, phagocytes, and endothelial cells in response to infection or tissue injury. Recent research has demonstrated that PTX3 activates lung fibroblasts to differentiate towards migrative and highly collagen-expressing myofibroblasts. Blocking PTX3 has been shown to attenuate lung injury-induced fibrosis [58]. Our bioinformatics analysis provides evidence suggesting that both SERPINE1 and PTX3 may serve as effective biomarkers of disease severity in IPF when assessed in lung tissue samples. However, further studies are needed to verify the roles of these two genes.

TSP-1 is produced by various cells and binds to components of the ECM. It plays a significant role in cellular processes such as wound healing, tissue repair, and fibrosis by activating latent TGF-β and stimulating collagen expression [10,59]. IHC staining in a previous study demonstrated positive TSP-1 staining in fibroblasts within the fibroblastic foci of the honeycomb areas of the IPF lung [60]. In our study, through integrated bioinformatics analysis using GEO datasets of bulk RNA profiling of lung fibroblasts isolated from IPF patients or bleomycin-treated mice, as well as single-cell RNA-seq of IPF patients and bleomycin-treated mice, we found evidence suggesting that TSP-1 is upregulated in lung fibroblasts during pulmonary fibrosis. This finding was further validated by our Western blot results. Additionally, analysis of three GEO datasets of TGF-β-treated lung fibroblasts also indicated the TGF-β-induced upregulation of TSP-1. Importantly, TSP-1 has been shown to activate TGF-β signaling and promote the pro-fibrotic phenotype of fibroblasts, thus contributing to the pathogenesis of cardiac, liver, and renal fibrosis [11,59,61]. In this study, we established stable TSP-1 overexpression in Mlg cells using lentiviral vectors and observed that TSP-1 overexpression alone promoted lung fibroblast activation, suggesting that the upregulation of fibroblast TSP-1 may contribute to fibroblast activation during pulmonary fibrosis.

Although the role of TSP-1 in fibrotic diseases has been extensively studied due to its activation of TGF-β, there has been limited research specifically focused on pulmonary fibrosis. Recently, Ruschkowski et al. demonstrated that LSKL treatment attenuated the upregulation of activated TGF-β1, phosphorylated Smad-3, and promoted interstitial macrophage infiltration in the lungs of neonatal rats exposed to bleomycin. In contrast, treatment with a TSP-1 mimic increases phosphorylated Smad-3, interstitial macrophage infiltration, and collagen content in the lung tissues of neonatal rats [62]. Consistent with their findings, our study revealed that intrapulmonary TSP-1 overexpression alone was sufficient to induce mild ER stress and pulmonary fibrosis, and it even exacerbated bleomycin-induced ER stress and pulmonary fibrosis. Mechanistically, TSP-1 promoted ER stress and fibroblast activation through CD47-dependent ROS production. Treatment with either LSKL or a CD47 inhibitor significantly attenuated BLM-induced ER stress and pulmonary fibrosis. These findings collectively suggest that the elevation of TSP-1 during pulmonary fibrosis is not merely a biomarker but likely plays a pathogenic role in the fibrotic changes in the lung.

Notably, a study conducted by Ezzie et al. [63] demonstrated that TSP-1 deficiency does not protect mice from bleomycin-induced pulmonary fibrosis, which contradicts our findings. This discrepancy is probably ascribed to the multifaceted functions and mechanisms of TSP-1 [64]. It is evident that TSP-1 deficient mice are susceptible to injury and inflammation, indicating the involvement of additional mechanisms beyond TGF-β activation. For instance, TSP-1 deficient mice exhibit an inability to effectively resolve neutrophilic inflammation and injury due to impaired IL-10 responses and suboptimal engagement of its receptor CD36 [15]. Interestingly, TSP-1-mediated resolution of lung inflammation is independent of TSP1-CD47 signaling or TGF-β activity [15]. In the present study, we employed intrapulmonary instillation of Lv-TSP-1 to restrict the local overexpression of TSP-1 in the lung. Treatment with LSKL specifically targeted the inhibitory effects on TSP-1 activity related to TGF-β1 activation. CD47 inhibitor also has limitations, given that TSP-1 binds to receptors other than CD47, and vice versa [64]. In fact, the function of TSP-1 is contextually defined by its interaction with various cell-surface receptors, including CD36, CD47, CD148, or integrins [64]. Therefore, future studies investigating the contribution of TSP-1 to fibroblast activation and pulmonary fibrosis should consider the involvement of TSP-1 receptors other than CD47.

The present study has two main limitations. Firstly, it focused on elucidating the predictive value of TSP-1 for the outcome of IPF patients. However, relying solely on a single biomarker for prognosis prediction may not be sufficient in the context of IPF. For instance, Li et al. established a hypoxia-immune-based prediction model for the prognosis of IPF patients [45]. Using the lasso regression method, they identified nine optimal variables from 29 hypoxia-immune-prognostic-related DEGs. These variables were then used to establish a multivariate Cox regression model to estimate the risk score for each patient. We acknowledge that TSP-1, as a univariate factor, may be less effective in predicting the outcome for IPF patients compared to these other univariate predictive models. The second limitation of this study was the absence of samples from human IPF patients, which hindered the validation of TSP-1 expression in human lung fibroblasts at the protein level.

## 5. Conclusions

This study presented novel findings regarding the correlation between UPR status and pulmonary function in patients with IPF. TSP-1 was upregulated in lung fibroblasts and identified as a potential biomarker for prognostic evaluation in IPF patients. TSP-1 overexpression alone was sufficient to induce mild ER stress and pulmonary fibrosis, and it could even exacerbate bleomycin-induced ER stress and pulmonary fibrosis. Mechanistically, TSP-1 promoted ER stress and fibroblast activation through CD47-dependent ROS production. Treatment with either a TSP-1 inhibitor or a CD47 inhibitor significantly attenuated BLM-induced ER stress and pulmonary fibrosis. Collectively, these findings suggest that the elevation of TSP-1 during pulmonary fibrosis is not merely a biomarker but likely plays a pathogenic role in the fibrotic changes in the lung.

## Data Availability

The data are available from the corresponding authors upon request.

## Supplementary Materials

The following supporting information can be downloaded at: https://www.mdpi.com/article/10.3390/antiox12122024/s1.
