# Supplementary material for: Investigation of a UPR-Related Gene Signature Identifies the Pro-Fibrotic Effects of Thrombospondin-1 by Activating CD47/ROS/Endoplasmic Reticulum Stress Pathway in Lung Fibroblasts
Source: Antioxidants (Basel). 2023 Nov 21;12(12):2024. doi: 10.3390/antiox12122024 (PMC10740656; doi:10.3390/antiox12122024)
Supplement: Supplementary file 1 [file antioxidants-12-02024-s001.zip › antioxidants-2679730-supplementary.pdf]

Investigation of a UPR-Related Gene Signature Identifies the Pro-Fibrotic Effects of Thrombospondin-1 by Activating CD47/ROS/Endoplasmic Reticulum Stress Pathway in Lung Fibroblasts

Jun-Hui Zhan [1,†], Juan Wei [1,2,†], Lin Liu [2], Yi-Tong Xu [1], Hui Ji [1], Chang-Nan Wang [3], Yu-Jian Liu [1,*] and Xiao-Yan Zhu [3,*]

[1] School of Kinesiology, The Key Laboratory of Exercise and Health Sciences of Ministry of Education, Shanghai University of Sport, Shanghai 200438, China
[2] School of Sports and Health, Nanjing Sport Institute, Nanjing 210014, China
[3] Department of Physiology, Navy Medical University, Shanghai 200433, China
\# These authors contributed equally to this work and should be considered as co-first authors
* Correspondence: liuyujian@sus.edu.cn (Y.L.); xiaoyanzhu@smmu.edu.cn (X.Z.);
Tel.: +86-021-65507533 (Y.L.);+86-021-81870984 (X.Z.)

Supplementary Materials and Methods

Administration of bleomycin and drug treatment

After anesthesia, the mice were intratracheally instilled with bleomycin (Selleck Chemicals, Houston, TX, USA) at a dose of 1.5 mg/kg in a total volume of 50 μl [1]. Control mice received only 50 μl of sterile PBS by endotracheal instillation. The TSP-1 inhibitor LSKL (MCE, New Jersey, USA) and CD47 inhibitor RRx-001 (MCE, New Jersey, USA) were prepared to the appropriate concentrations and stored at -80°C for subsequent experiments after sequential addition of 10% DMSO and 90% corn oil according to the reagent vendor's requirements. To investigate the role of LSKL or RRx-001 in bleomycin-induced lung fibrosis, mice were randomly divided into the following groups: (1) Control group: mice were intratracheally instilled with PBS and injected intraperitoneally with vehicle. (2) LSKL group: mice were intratracheally instilled with PBS and injected intraperitoneally with 4 mg/kg LSKL [2]. (3) RRx-001 group: mice were intratracheally instilled with PBS and injected intraperitoneally with 10 mg/kg RRx-001 [3, 4]. (4) Bleomycin Group: mice were intratracheally instilled with bleomycin and injected intraperitoneally with vehicle. (5) Bleomycin and LSKL: mice were intratracheally instilled with bleomycin and injected intraperitoneally with 4 mg/kg LSKL. (6) Bleomycin and RRx-001: mice were intratracheally instilled with bleomycin and injected intraperitoneally with 10 mg/kg RRx-001. LSKL, RRx-001 or vehicle was injected on day 2 after the instillation of bleomycin. LSKL was daily injected, and RRx-001 treatment was continued every other day until the end of the experiment. Lung samples were collected 14 days after bleomycin instillation.

TSP-1-expressing lentiviral vector (Lv-TSP-1) and control lentiviral vector (Lv-TSP-1) were purchased from Genechem Co., Ltd. (Shanghai, China). After anesthesia, ICR mice were intratracheally instilled with 4×10^7 PFUs (in 50 μL) of Lv-TSP-1 or Lv-TSP-1 [5, 6]. Forty-eight hours later, a single dose of bleomycin was administered intratracheally at a dose of 1.5 mg/kg in a total volume of 50 μL. To investigate the role of intrapulmonary TSP-1 overexpression in bleomycin-induced pulmonary fibrosis, the mice were randomly divided into 4 groups: (1) Lv-Control group: mice were intratracheally instilled with Lv-Control and PBS; (2) Lv-TSP-1 group: mice were intratracheally instilled with Lv-TSP-1 and PBS; (3) Lv-Control + bleomycin group: mice were instilled with bleomycin and Lv-Control; (4) Lv-TSP-1 + bleomycin group: mice were intratracheally instilled with Lv-TSP-1 and bleomycin. Lung samples were collected 14 days after bleomycin instillation.

Identification of UPR Status and differentially expressed genes (DEGs)

The reference gene sets for the unfolded protein response (UPR) were downloaded from the Molecular Signatures Database (MSigDB, HALLMARK_UNFOLDED_PROTEIN_RESPONSE.v2022.1.Hs.json) which include 113 genes. The Uniform Manifold Approximation and Projection (UMAP) algorithm, a non-linear dimensionality reduction algorithm, was applied to divide the samples into two clusters. Based on these clusters, two groups, "UPR^{high}" and "UPR^{low}", were identified. The limma algorithm was applied to identify DEGs between the two groups. The "Benjamini & Hochberg" was used to adjust the *p*-values for multiple testing. Genes with a *p*-value < 0.05 and an absolute value of log2 (fold change) > 1 were considered as UPR-related DEGs.

Quantitative Polymerase Chain Reaction (qPCR)

Total RNA was extracted from tissues and cells using TRIzol reagent (Invitrogen) according to the manufacturer's instructions. Samples were reverse transcribed to complementary DNA (cDNA) using the PrimeScript® RT reagent Kit with gDNA Eraser (Takara, Dalian, China). qPCR was performed using ChamQ Universal SYBR qPCR Master Mix (Vazyme, China). Relative mRNA expression levels were quantified using the $2^{-\Delta\Delta Ct}$ method. Primers used for qPCR were synthesized by Sangon Biotech Co., Ltd. The primer sequences are provided in Supplemental Table S1.

Cell culture and stable transfection

Mouse lung fibroblast Mlg cells were purchased from Jennio Biotech Co. (Guangzhou, China) and cultured in DMEM/F12 (Gibco, USA) medium containing 10% FBS (Gibco, USA) and 1% penicillin/streptomycin (Gibco, USA). Mlg cells were transfected with lentivirus vector expressing TSP-1 (Lv-TSP-1) or control lentivirus (Lv-Control), which were designed and synthesized by Shanghai Genetic Chemical Co. After 72 h of transfection, Mlg cells were treated with 1 μg/ml puromycin (MCE, USA) to generate a stable TSP-1-overexpressing cell line [7]. The overexpression efficiency of Lv-TSP-1 was confirmed by quantitative polymerase chain reaction and Western blot analysis.

Western blot

Total proteins were extracted from lung tissues, primary fibroblasts, and Mlg cells using cold RIPA buffer containing a protease and phosphatase inhibitor cocktail (Proteintech, Wuhan, China). After electrophoresis, the proteins were transferred to PVDF membranes (Millipore, Massachusetts, USA). The membranes were blocked in 5% (w/v) non-fat skimmed milk/TBST for 1.5 hours. Primary antibodies against α-SMA (1:500, Servicebio), Fibronectin (1:1000, Cell Signaling Technology, Massachusetts, USA), Grp78 (1:1000, Cell Signaling Technology), CHOP (1:1000, Cell Signaling Technology), TSP-1 (1:1000, Proteintech), and β-actin (1:3000, Sigma, Missouri, USA) were incubated with the membranes overnight at 4 °C. After washing three times with TBST buffer, the membranes were incubated with horseradish peroxidase-labeled secondary antibodies (1:1000, Proteintech) for 1 hour at room temperature. The Western blots were subsequently detected using enhanced chemiluminescence (Biosharp, Hefei, China), and the chemiluminescent signals were quantified using the Tanon Chemiluminescence Instrument (Tanon, Shanghai, China). The intensity of the strips was quantified using ImageJ software.

Statistical Analysis

The R software (version 4.2.0) and the corresponding packages were used for the bioinformatic analysis. The general idea and methodologies used in the bioinformatic analysis were shown in a flow chart (Figure 1). The packages "ggplot2" and "pheatmap" were utilized to optimize data presentation. The data were presented as means ± SEM. Raw data were analyzed for normal distribution using the Kolmogorov–Smirnov test. If the data were normally distributed, they were compared using two-tailed unpaired t-tests or one-way analysis of variance (ANOVA), followed by a Student–Newman–Keuls post hoc test for two-group or multi-group comparisons, respectively. A p value of < 0.05 was considered significant.

References

1. Li, S.R.; Tan, Z.X.; Chen, Y.H.; Hu, B.; Zhang, C.; Wang, H.; Zhao, H.; Xu, D.X. Vitamin D deficiency exacerbates bleomycin-induced pulmonary fibrosis partially through aggravating TGF-β/Smad2/3-mediated epithelial-mesenchymal transition. *Respir Res* **2019**, 20, 266.
2. Krishna, S.M.; Seto, S.W.; Jose, R.J.; Biros, E.; Moran, C.S.; Wang, Y.; Clancy, P.; Golledge, J. A peptide antagonist of thrombospondin-1 promotes abdominal aortic aneurysm progression in the angiotensin II-infused apolipoprotein-E-deficient mouse. *Arterioscler Thromb Vasc Biol* **2015**, 35, 389-98.
3. Niu, S.; Cheng, K.; Jia, L.; Liang, J.; Mu, L.; Wang, Y.; Yang, X.; Yang, C.; Zhang, Y.; Wang, C.; et al. Lineage tracing of mutant granulosa cells reveals in vivo protective mechanisms that prevent granulosa cell tumorigenesis. *Cell Death Differ* **2023**, 30, 1235-1246.
4. Chen, Y.; He, H.; Lin, B.; Chen, Y.; Deng, X.; Jiang, W.; Zhou, R. RRx-001 ameliorates inflammatory diseases by acting as a potent covalent NLRP3 inhibitor. *Cell Mol Immunol* **2021**, 18, 1425-1436.
5. Wei, J.; Zhan, J.; Ji, H.; Xu, Y.; Xu, Q.; Zhu, X.; Liu, Y. Fibroblast Upregulation of Vitamin D Receptor Represents a Self-Protective Response to Limit Fibroblast Proliferation and Activation during Pulmonary Fibrosis. *Antioxidants (Basel)* **2023**, 12.
6. Guo, L.; Li, S.; Zhao, Y.; Qian, P.; Ji, F.; Qian, L.; Wu, X.; Qian, G. Silencing Angiopoietin-Like Protein 4 (ANGPTL4) Protects Against Lipopolysaccharide-Induced Acute Lung Injury Via Regulating SIRT1 /NF-kB Pathway. *J Cell Physiol* **2015**, 230, 2390-402.
7. Yang, F.; Liu, Y.; Tu, J.; Wan, J.; Zhang, J.; Wu, B.; Chen, S.; Zhou, J.; Mu, Y.; Wang, L. Activated astrocytes enhance the dopaminergic differentiation of stem cells and promote brain repair through bFGF. *Nat Commun* **2014**, 5, 5627.

Supplementary tables

Table S1: The primer sequences used in this research.

Primer	The gene accession number	Forward (5′→3′)	Reverse (3′→5′)
β-actin	NM_007393.5	CACGATGGAGGGGCCGGACTCATC	TAAAGACCTCTATGCCAACACAGT
TSP-1	NM_001313914.1	GGGGAGATAACGGTGTGTTTG	CGGGGATCAGGTTGGCATT
Fibronectin	NM_010233.2	CAACAACCGGAATTACACC	GGTCTCGGAGCTGGGAGTAG
Collagen-1	NM_007742.4	GACATGTTCAGCTTTGTGGACCTC	GGGACCCTTAGGCCATTGTGTA
Nox1	NM_172203.2	CGTGAAAAGATGACCCAGATCA	TGGTACGACCAGAGGCATACAG
Nox2	NM_007807.5	CCAACTGGGATAACGAGTTCAA	TCAGGGCCACACAGGAAAA
Nox3	NM_198958.2	GCACCGGGACAGTACATCTT	AGTGACTCCAATTCCCGTTG
Nox4	NM_015760.5	CCGGACAGTCCTGGCTTATC	TGCTTTTATCCAACAATCTTCTTTT
Nox5	NM_008898.2	TGCCAGTGTGATCTATCTGCT	TCGGCTTCTTTCGGACCTCT

Table S2: Basic information of IPF patients in discovery cohort.

Characteristics	Options	Whole cohort (119)
Gender	Male	64 (0.538)
	Female	55 (0.462)
Age	≥65 years	53 (0.445)
	<65 years	66 (0.555)
Smoking history	Former	70 (0.588)
	Nonsmorker	41 (0.345)
	Unknown	8 (0.067)

Table S3: Basic information of the UPR^{low} and UPR^{high} clusters.

Characteristics	Whole cohort (119)	UPR^{low} (80)	UPR^{high} (39)
Gender			
Male	64 (0.538)	51 (0.638)	13 (0.333)
Female	55 (0.462)	29 (0.362)	26 (0.667)
Age			
≥65 years	53 (0.445)	41 (0.512)	12 (0.308)
<65 years	66 (0.555)	39 (0.488)	27 (0.692)
Smoking history			
Former	70 (0.588)	47 (0.588)	23 (0.590)
Nonsmorker	41 (0.345)	29 (0.362)	12 (0.308)
Unknown	8 (0.067)	4 (0.050)	4 (0.102)

Table S4: Correlation coefficient and significance of DEGs.

Genes	SGRQ	FVC	DLCO
TNFAIP6	0.36635 **	-0.44951 **	-0.37605 **
THBS1	0.39467 **	-0.56261 **	-0.48151 **
SPP1	0.11131	-0.25864 *	-0.15317
SOSTDC1	-0.1531	0.24139 *	0.18896
SLC7A5	0.26198 *	-0.44821 **	-0.36719 **
SERPINE1	0.44255 **	-0.52398 **	-0.42882 **
SERPINA3	0.44943 **	-0.53019 **	-0.41905 **
SELE	0.39628 **	-0.43678	-0.49731 **
RTKN2	-0.17881	0.35167 **	0.2807 *
RGS1	0.30607 *	-0.33104 **	-0.31429 *
PTX3	0.39268 **	-0.51742 **	-0.4331 **
MYC	0.27663 *	-0.41524 **	-0.33983 **
MT1M	0.28398 *	-0.34146 **	-0.27379 *
MMP1	0.17066	-0.32572 **	-0.27452 *
LTF	0.07686	-0.22492	-0.07319
IL6	0.2527 *	-0.39732 **	-0.30495 *
IL1R2	0.39145 **	-0.51228 **	-0.45114 **
IGKV6-21	-0.10767	-0.04316	0.06184
IGKV5-2	0.00168	-0.08601	0.01442
IGKV1D-27	-0.08256	-0.15376	0.03438
IGHV1OR15-1	-0.04319	-0.19239 *	-0.13372
HP	0.26798 *	-0.40224 **	-0.07671
GFPT2	0.12885	-0.38533 **	-0.18232
DDIT4	0.28135 *	-0.31244 **	-0.26645 *

CYP24A1	0.15456	-0.24065 *	-0.20836 *
CXCL13	0.01998	-0.23223 *	-0.09782
CCL20	0.28002 *	-0.40424 **	-0.23114 *
BPIFA1	0.06644	-0.1489	-0.03473
ADAMTS4	0.39872 **	-0.51023 **	-0.45018 **
ADAMTS1	0.34673 **	-0.3868 **	-0.35605 **

* $p < 0.05$, ** $p < 0.01$.

Table S5: Basic information of patients in discovery cohort and validation cohort.

Accession	Platform	Diagnosis			Gender			Age	
		Control	IPF	Other	Male	Female	Unknow	<65 years	≥65 years
GSE32537 (217)	GPL6244	50 (0.23)	119 (0.54)	48 (0.22)	129 (0.60)	88 (0.40)	0 (0)	145 (0.67)	72 (0.33)
GSE27957 (45)	GPL5175	0 (0)	45 (1)	0 (0)	40 (0.889)	5 (0.111)	0 (0)	28 (0.622)	17 (0.378)
GSE38958 (115)	GPL5175	45 (0.391)	70 (0.609)	0 (0)	85 (0.739)	30 (0.261)	0 (0)	33 (0.287)	82 (0.713)
GSE47460 (268)	GPL6480	17 (0.309)	38 (0.691)	0 (0)	38 (0691)	17 (0.309)	0 (0)	32 (0.582)	23 (0.418)
	GPL14550	91 (0.427)	122 (0.573)	0 (0)	121 (0.568)	92 (0.432)	0 (0)	100 (0.469)	113 (0.531)
GSE93606 (77)	GPL11532	20 (0.260)	57 (0.740)	0 (0)	50 (0.649)	27 (0.351)	0 (0)	30 (0.390)	47 (0.610)
GSE135893 (30)	GPL18573<br>GPL20301<br>GPL24676	10 (0.333)	20 (0.667)	0 (0)	20 (0.667)	9 (0.300)	1 (0.033)	20 (0.667)	10 (0.333)
GSE132771 (8)	GPL24676	3 (0.375)	3 (0.375)	2 (0.250)	NA	NA	NA	NA	NA
GSE119007 (11)	GPL20301	6 (0.545)	5 (0.455)	0 (0)	6 (0.545)	5 (0.455)	0 (0)	NA	NA
GSE135097 (9)	GPL13534	4 (0.444)	5 (0.556)	0 (0)	6 (0.667)	3 (0.333)	0 (0)	NA	NA
GSE226249 (14)	GPL20301	4 (0.286)	10 (0.714)	0 (0)	10 (0.714)	4 (0.286)	0 (0)	7 (0.500)	7 (0.500)

NA, data not available.

Supplementary figures

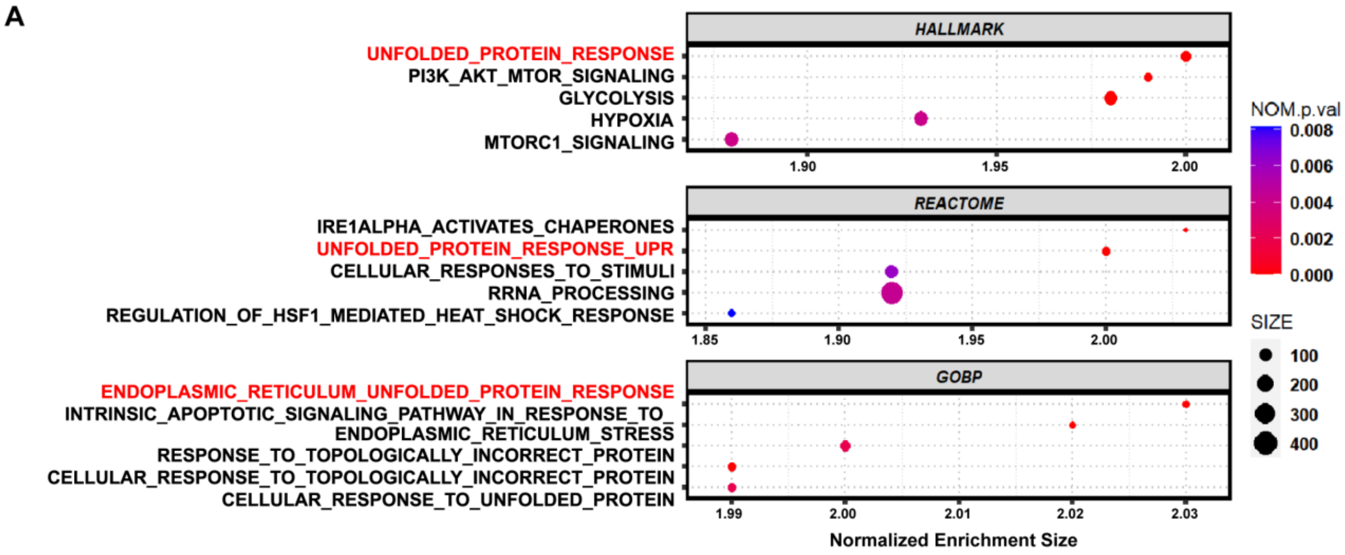

Supplementary Figure S1. A high UPR status is associated with poorer pulmonary function in patients with IPF. (A) Intercept the top five most significant pathways in the result of HALLMARK, REACTOME and GOBP datasets, respectively. UPR signaling was found to be common among the top three gene sets identified by GSEA. The size of the dot represents the number of genes in the pathway that are included in the expression data set, and the color represents the *p*-value.

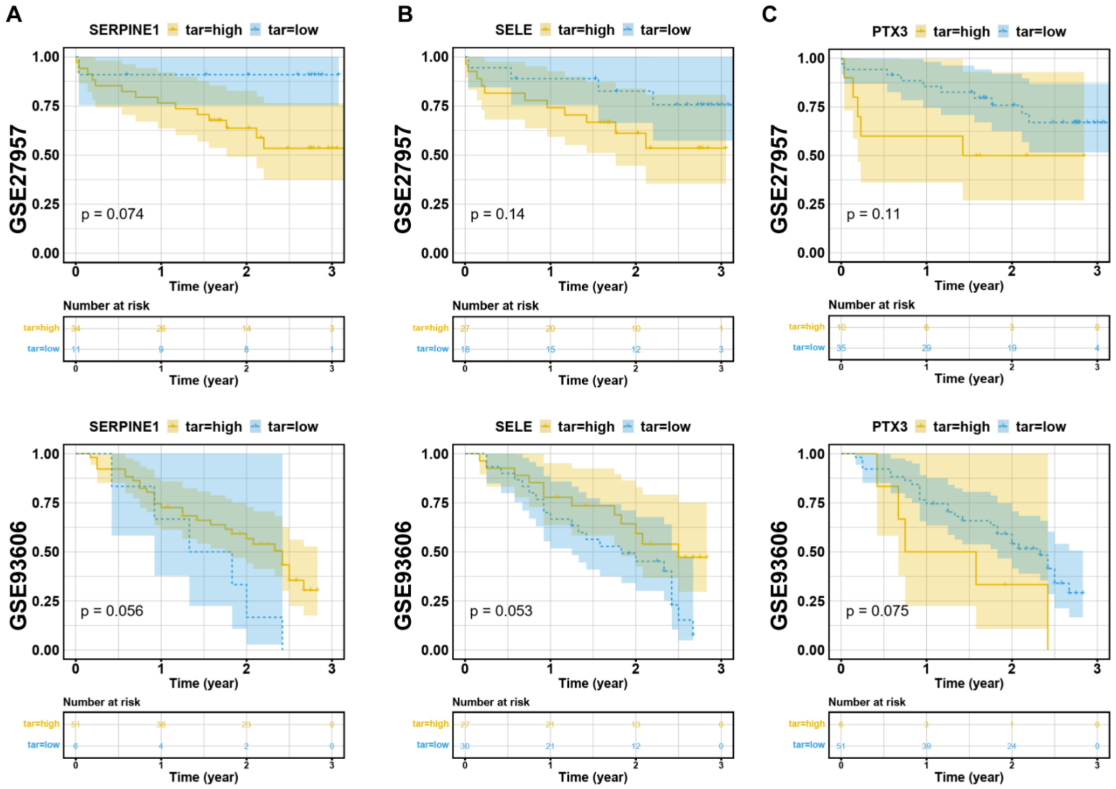

Supplementary Figure S2. Survival analysis of the partial validation cohort on the five genes to be selected. (A) Kaplan-Meier plot of overall survival between high level SERPINE1 and low level SERPINE1 expression in IPF cohorts of GSE27957 (top) and GSE93606 (bottom). (B) Kaplan-Meier plot of overall survival between high level SELE and low level SELE expression in IPF cohorts of GSE27957 (top) and GSE93606 (bottom). (C) Kaplan-Meier plot of overall survival between high level PTX3 and low level PTX3 expression in IPF cohorts of GSE27957 (top) and GSE93606 (bottom). The cutoff value is selected by survminer package.

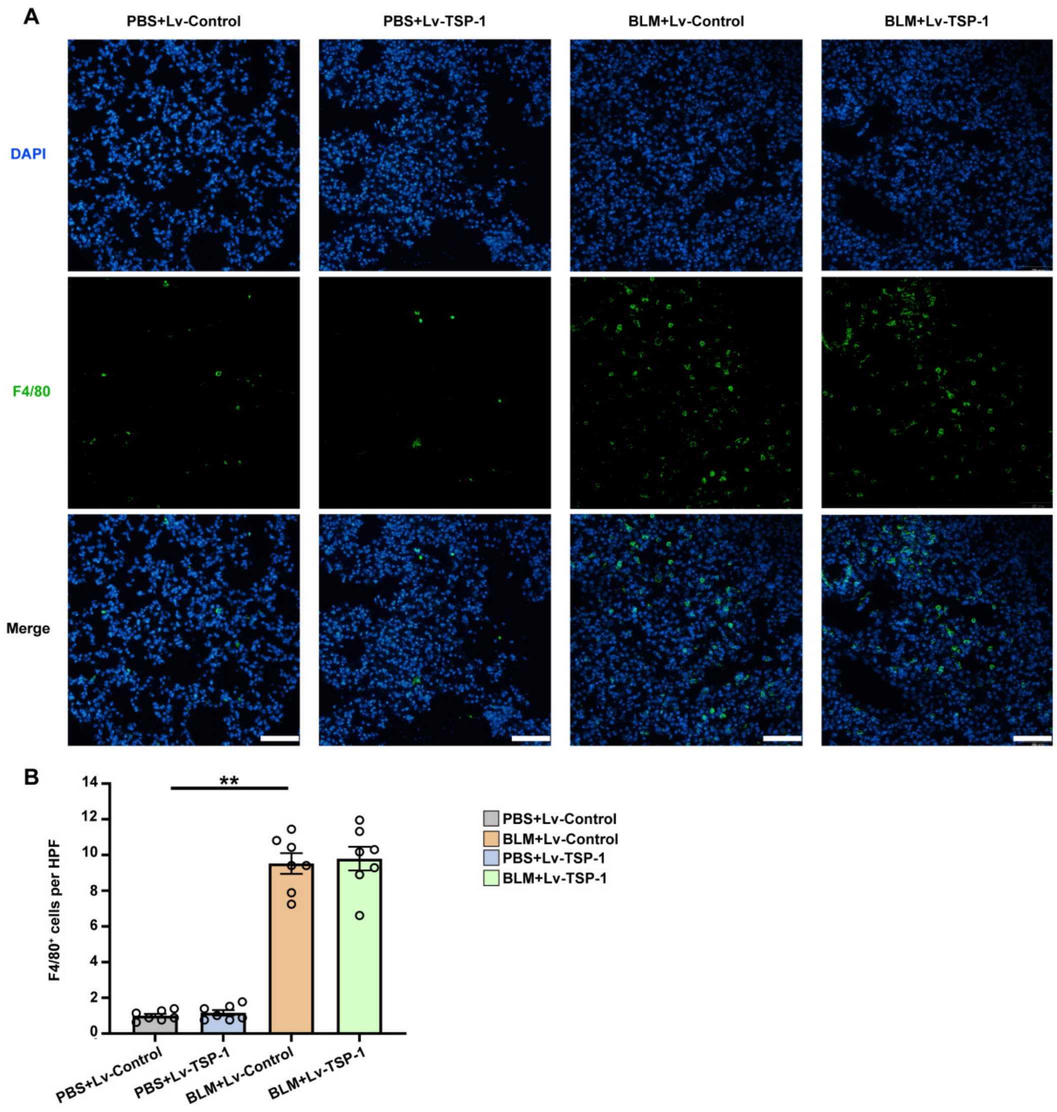

Supplementary Figure S3. The effect of intrapulmonary TSP-1 overexpression on macrophage infiltration in bleomycin-treated mice. Mice were randomly divided into four groups: Lv-Control, Lv-TSP-1, Lv-Control + BLM, and Lv-TSP-1 + BLM. (A) Macrophage infiltration in lung tissues were examined by immunofluorescence staining of macrophage marker F4/80 (Alexa Fluor 488, green). The nuclei were stained blue by DAPI, and the images were taken at ×200 magnification. Scale bars correspond to 50 μm. (B) Quantification of F4/80+ staining cells per high-power field (HPF). Data are expressed as mean ± SEM (n = 7). ** $p < 0.01$. BLM represents bleomycin.

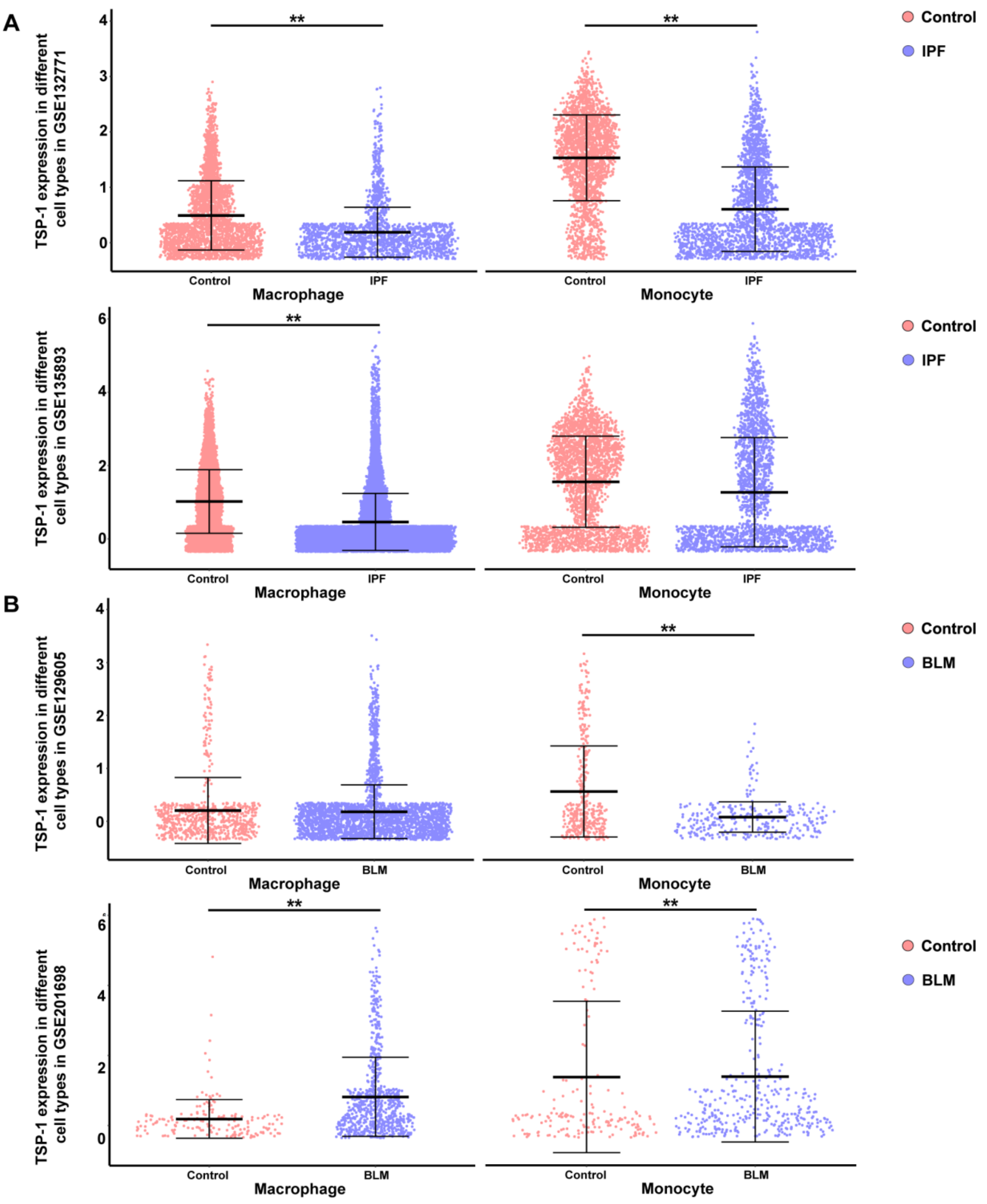

Supplementary Figure S4. TSP-1 expression in macrophages and monocytes in lung tissues of IPF patients and murine models of bleomycin-induced pulmonary fibrosis. Single-cell RNA sequencing demonstrated the expression profile of TSP-1 in Macrophage (left) and Monocyte (right) in lung tissues of IPF patients (A) and murine models of bleomycin-induced pulmonary fibrosis (B). ** $p < 0.01$. BLM represents bleomycin.

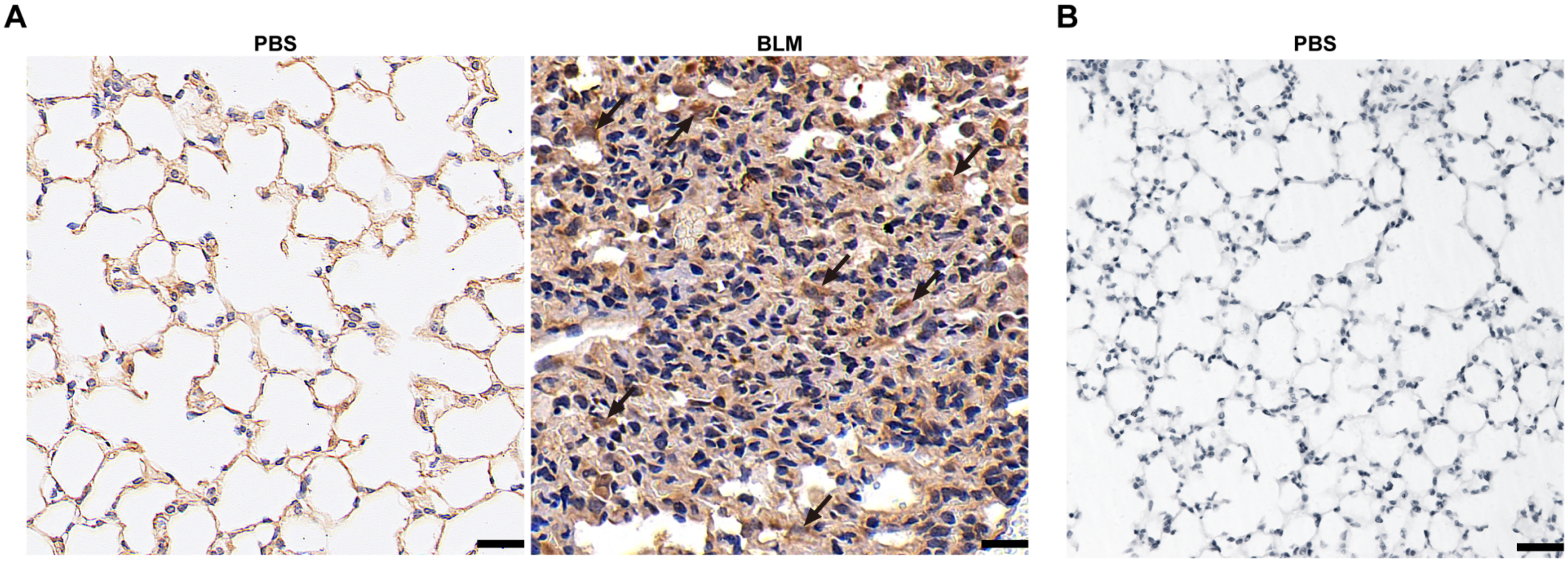

Supplementary Figure S5. TSP-1 is upregulated in lung tissues during pulmonary fibrosis. Mice were intratracheally instilled with bleomycin (1.5 mg/kg) or PBS. Lung tissues were obtained at day 14 after instillation of bleomycin or PBS and used for immunohistochemistry staining of TSP-1 (A). Black arrows point to TSP-1 positive stained fibroblasts within fibroblastic foci. For negative controls, the primary antibody was substituted with a mouse IgG in the same dilution (B). Scale bars correspond to 20 μm. BLM represents bleomycin.

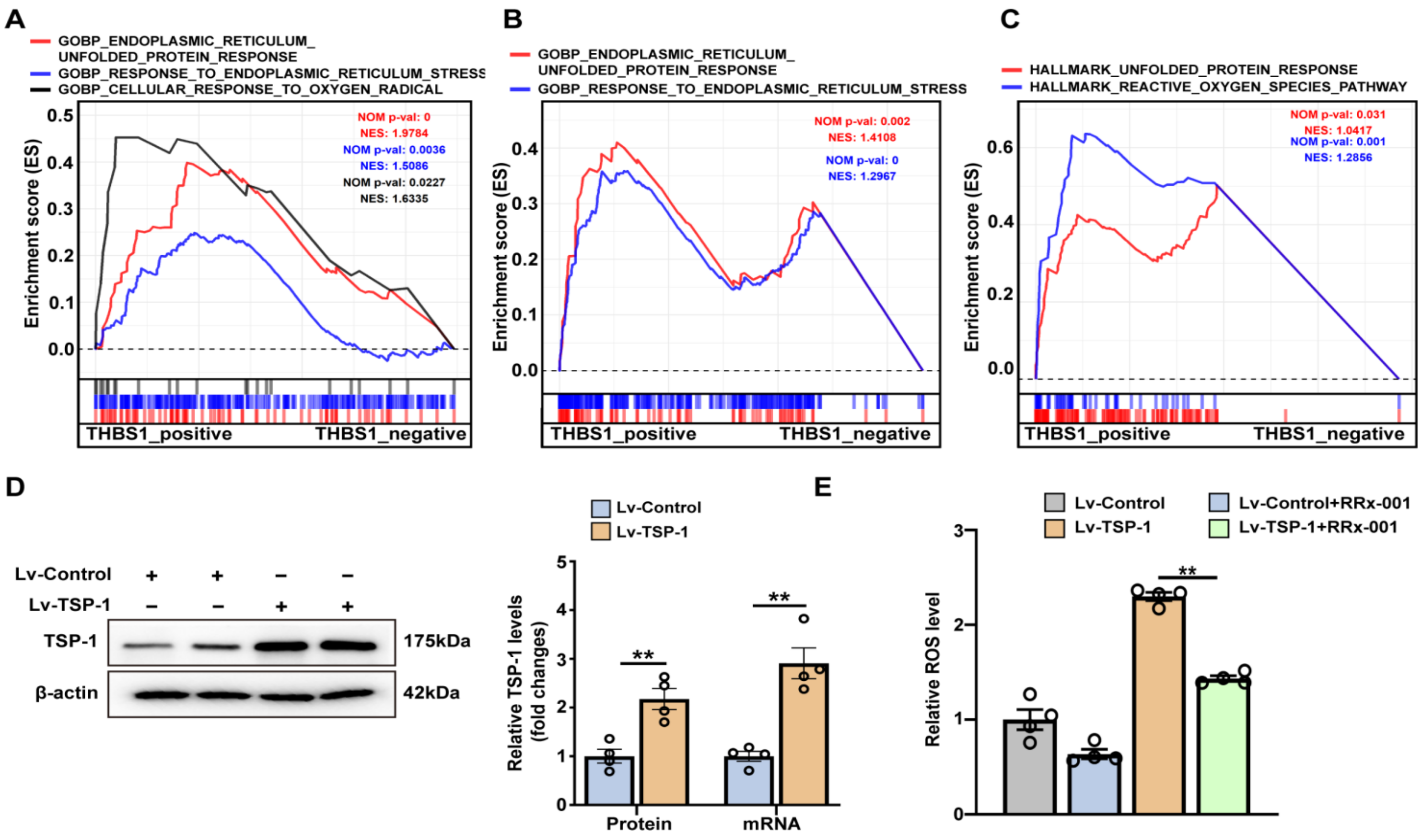

Supplementary Figure S6. Stable overexpression of TSP-1 promotes fibroblast activation by CD47/ROS/ER stress signaling pathway (related to Figure 7). The GSEA analysis of selecting TSP-1 expression level as phenotype. Following reference datasets HALLMARK and GOBP, the expression level of TSP-1 is positively correlated with UPR, ROS and ERs pathway in GSE97826 (A), GSE97829 (B) and GSE172121 (C). (D) The overexpression efficiency of stable transfection of Lv-TSP-1 or Lv-Control was confirmed by measuring mRNA and protein levels of TSP-1 by qPCR and Western blot analysis, respectively. Data are expressed as mean ± SEM (n = 4). $^{**}p < 0.01$.

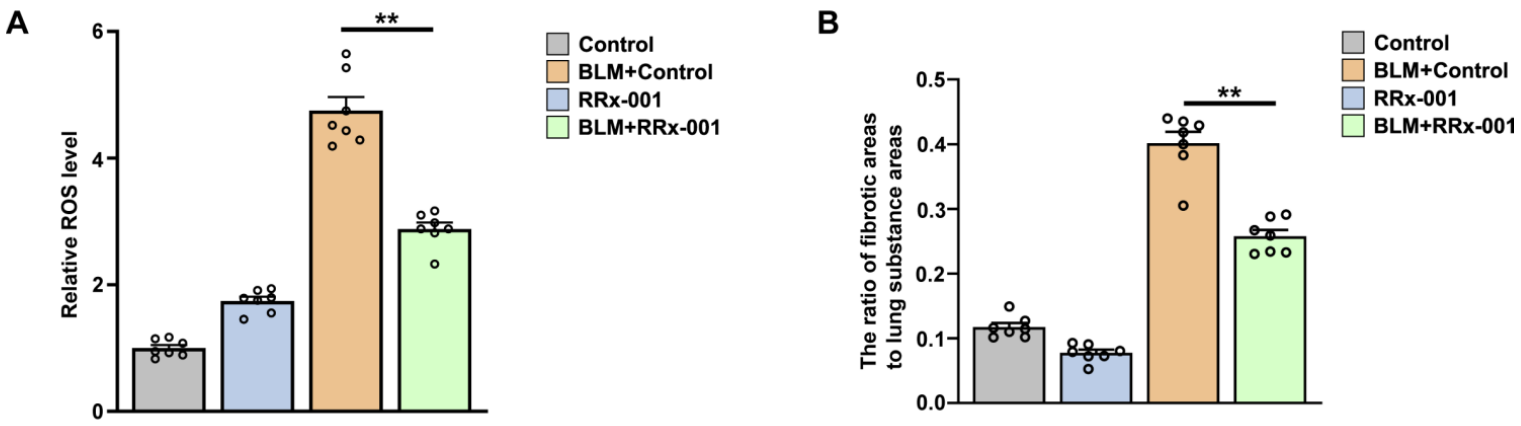

Supplementary Figure S7. CD47 inhibitor attenuates BLM-induced ROS production, ER stress and pulmonary fibrosis (related to Figure 8). (A) Quantification of the ROS production in fibroblasts in the Control, RRx-001, BLM + Control, and BLM + RRx-001 groups. (B) Quantification of the ratio of collagen-deposited areas to lung substance areas (a morphometric measure of pulmonary fibrosis). Data are expressed as mean ± SEM (n = 7). $^{**}p < 0.01$. BLM represents bleomycin.
